# Realization of highly asymmetric hydrogenated graphene in the van der Waals confined space

**DOI:** 10.1093/nsr/nwaf067

**Published:** 2025-02-22

**Authors:** Xianlei Huang, Hang Zheng, Weilin Liu, Li Zhu, Guowen Yuan, Jie Xu, Kaiyuan Wang, Lei Wang, Shao-Chun Li, Libo Gao

**Affiliations:** National Laboratory of Solid State Microstructures, Jiangsu Key Laboratory for Nanotechnology, School of Physics, Collaborative Innovation Center of Advanced Microstructures, Nanjing University, Nanjing 210008, China; National Laboratory of Solid State Microstructures, Jiangsu Key Laboratory for Nanotechnology, School of Physics, Collaborative Innovation Center of Advanced Microstructures, Nanjing University, Nanjing 210008, China; National Laboratory of Solid State Microstructures, Jiangsu Key Laboratory for Nanotechnology, School of Physics, Collaborative Innovation Center of Advanced Microstructures, Nanjing University, Nanjing 210008, China; National Laboratory of Solid State Microstructures, Jiangsu Key Laboratory for Nanotechnology, School of Physics, Collaborative Innovation Center of Advanced Microstructures, Nanjing University, Nanjing 210008, China; National Laboratory of Solid State Microstructures, Jiangsu Key Laboratory for Nanotechnology, School of Physics, Collaborative Innovation Center of Advanced Microstructures, Nanjing University, Nanjing 210008, China; National Laboratory of Solid State Microstructures, Jiangsu Key Laboratory for Nanotechnology, School of Physics, Collaborative Innovation Center of Advanced Microstructures, Nanjing University, Nanjing 210008, China; Anhui Provincial Key Laboratory of Magnetic Functional Materials and Devices, School of Materials Science and Engineering, Anhui University, Hefei 230601, China; National Laboratory of Solid State Microstructures, Jiangsu Key Laboratory for Nanotechnology, School of Physics, Collaborative Innovation Center of Advanced Microstructures, Nanjing University, Nanjing 210008, China; National Laboratory of Solid State Microstructures, Jiangsu Key Laboratory for Nanotechnology, School of Physics, Collaborative Innovation Center of Advanced Microstructures, Nanjing University, Nanjing 210008, China; National Laboratory of Solid State Microstructures, Jiangsu Key Laboratory for Nanotechnology, School of Physics, Collaborative Innovation Center of Advanced Microstructures, Nanjing University, Nanjing 210008, China; National Laboratory of Solid State Microstructures, Jiangsu Key Laboratory for Nanotechnology, School of Physics, Collaborative Innovation Center of Advanced Microstructures, Nanjing University, Nanjing 210008, China

**Keywords:** van der Waals confined space, highly asymmetric, hydrogenated graphene, proton

## Abstract

The van der Waals (vdW) confined space provides a distinct environment from free space, enabling the production of two-dimensional Janus materials, like highly asymmetric hydrogenated graphene (AH-Gr). Here, we develop a vdW confined space assisted hydrogenation method to produce AH-Gr. The confined space between graphene and the substrate aggregates hydrogen radicals, making the bottom-side of graphene more prone to hydrogenation. The dense and homogeneous confined spaces between adjacent vdW crystals promote rapid and uniform distribution of carbon-hydrogen (C−H) bonds. The hydrogen-to-carbon atomic (H/C) ratios can be quantitatively controlled by adjusting the permeated proton dose. All AH-Gr, regardless of H/C ratios, remain vacancy-free. The spatial distributions of C−H bonds significantly influence the electrical and magnetic properties of AH-Gr. Asymmetric hydrogenation transforms graphene from a semi-metal to a semiconductor, suppresses the quantum Hall effect, and reduces the phase coherence length. This study provides new insights into the preparation and characteristics of hydrogenated graphene, broadening the applications of vdW confined space.

## Teaser

This study leverages confined spaces to achieve selective hydrogenation of graphene through permeating protons, producing highly structurally asymmetric hydrogenated graphene, offering novel insights into the fabrication and properties of hydrogenated graphene.

## INTRODUCTION

Van der Waals (vdW) confined space, the empty space between adjacent vdW crystals spanning angstroms to nanometers [1], provides a fantastic nano-environment of causing various physical and chemical performances, including the vdW pressure [2,3], frictionless flow [4], untraditional phase transition [5,6], selective permeation [7,8], confined catalysis [9], *etc*. The narrowest interlayer space restricts the transport of most molecules and ions [10], but they can be expanded by intercalating alkalis and acid molecules under specific conditions [11,12]. The spaces between vdW crystals and the non-vdW substates are also regarded as vdW confined spaces [5], albeit less homogeneous but still prevalent and significantly influential. For example, water or oxygen molecules confined between graphene and SiO_2_/Si can induce *p*-type doping of graphene [13].

Graphene, the first exfoliated vdW crystal, holds promise in electronic, photonic and spintronic applications [14]. However, its lack of an intrinsic bandgap restricts its use in transistors and sensors [15]. Bandgaps can be induced by breaking the graphene's structural symmetry through functionalization with various molecules [16,17], such as hydrogen [18–22], fluorine [23], organic radicals [16], *et al*. Among these, hydrogenation is widely employed due to its accessibility, reversibility and harmlessness [18,21]. Depending on the distribution of carbon-hydrogen (C−H) bonds, hydrogenated graphene can be classified into lowly asymmetric forms (LH-Gr), such as ideal double-side hydrogenated graphene, and highly asymmetric forms (AH-Gr), such as ideal single-side hydrogenated graphene [18,24]. AH-Gr exhibits distinct properties compared to intrinsic graphene [25], including an indirect bandgap [26,27], strong spin-orbit coupling [28,29], spin-triplet exciton [30] and ferromagnetism [31]. Particularly, graphone, a fully hydrogenated form of single-side hydrogenated graphene, is theoretically predicted to manifest many distinctive properties [29,31].

Currently, hydrogenation of graphene typically utilizes hydrogen radicals or protons as reactants, including H_2_ plasma [18,21], hydrogen atom exposure [19,20,22,32] and hydrogen silsesquioxane dissociation [28]. AH-Gr films have been reported to be produced by treating graphene on SiO_2_/Si (Gr/SiO_2_) substrate with hydrogen plasma [18], generating active H radicals on epitaxial graphene under ultra-high vacuum (UHV) [32], or applying hydrogen silsesquioxane followed by electron beam irradiation [28]. These methods introduce magnetic moment asymmetry and significant spin-orbit coupling in hydrogenated graphene [28,32]. However, recent findings indicate that the graphene's hexagonal lattices are permeable to both protons and hydrogen molecules [33–36], complicating the construction of AH-Gr since both sides are exposed to active H radicals or protons. Therefore, achieving AH-Gr with a precise hydrogen-to-carbon atomic ratio (H/C) remains a significant challenge.

The crucial strategy for producing AH-Gr lies in designing differentiated chemical environments on each side of the graphene. Actually, graphene on substrate fulfills this criterion, i.e. free space on the upper side and confined space on the bottom. The vdW confined space between graphene and substrate serves as a nano-sized reactive chamber that influences internal chemical reactions [5,9], enhancing the stability of active sites and improving reaction activities [37]. In this study, we propose a vdW confined space assisted hydrogenation method for precise AH-Gr production. The permeated protons accumulate and recombine into H radicals between graphene and substate, resulting in a higher hydrogen concentration that facilitates hydrogenation at the bottom side. By controlling the stabilization conditions of C−H bonds, the bottom side of graphene can be controllably hydrogenated, while the upper side remains almost non-hydrogenated. Comprehensive characterizations confirm the highly asymmetreic feature and the recoverability of graphene lattices. The degree of hydrogenation in AH-Gr can be precisely controlled by adjusting the dose of permeated protons. The spatial distributions of hydrogen atoms significantly influence electrical and magnetic properties, allowing AH-Gr transformation from semi-metal to semiconductor even at low ppm hydrogenation levels.

## RESULTS AND DISCUSSION

### AH-Gr in the vdW confined space

**Figure 1.**
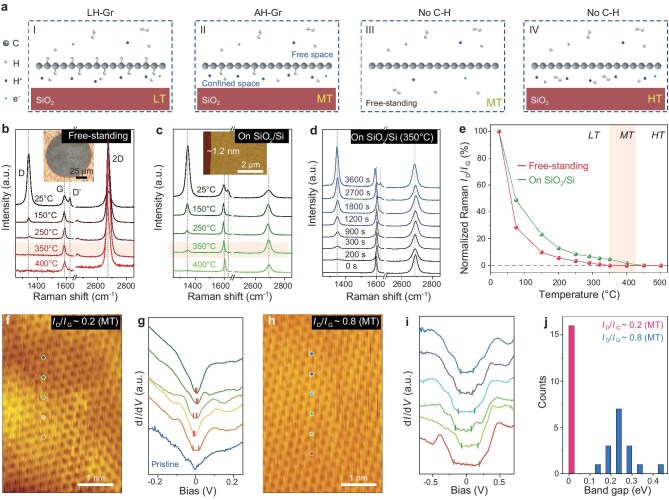
Production of AH-Gr in the vdW confined space via proton permeation. (a) Schematics of hydrogenating graphene through different treatment processes. I, Gr/SiO_2_ is exposed to H_2_ plasma at LT, where protons can permeate graphene and form LH-Gr with the C−H bonds on both sides. II, Gr/SiO_2_ is exposed to H_2_ plasma at MT, where C−H bonds are formed mainly on bottom side due to the high-density H radicals within the vdW confined space lead to AH-Gr. III and IV, no C−H bonds are formed for free-standing graphene at MT (III) and Gr/SiO_2_ at HT (IV). (b, c) Raman spectra of free-standing graphene (b) and Gr/SiO_2_ (c) hydrogenated at different temperatures for 300 s. The hydrogenation temperature of 350°C is highlighted by light yellow, where Gr/SiO_2_ is hydrogenated but free-standing graphene is not. Insets show the corresponding optical image of free-standing graphene on Cu grid (b) and AFM image of Gr/SiO_2_ (c). (d) Raman spectra of hydrogenating Gr/SiO_2_ at 350°C with different durations. The intensity of D peak increases with prolonging the proton permeation time. (e) Normalized Raman *I*_D_/*I*_G_ ratios of free-standing graphene and Gr/SiO_2_ hydrogenated at different temperatures. The ratios of *I*_D_/*I*_G_ correspond to the relative concentration of C−H bonds, and LT is thereby defined as the temperatures below 350°C, MT is 350–425°C labelled by light yellow, and HT is above 425°C. (f, h) Typical STM topographical images of AH-Gr/SiO_2_ with *I*_D_/*I*_G_ of 0.2 (f) and 0.8 (h). There are no obvious atomic defects or hydrogen atoms chemisorbed on the upper side. (g, i) Corresponding STS spectra of (f) and (h) collected at the colored dots. The STS spectrum of pristine graphene is shown as the blue line in (g) for comparison. (j) Distribution histogram of STS bandgaps from (g) and (i). Their bandgaps range from 0 to 30 meV for AH-Gr with *I*_D_/*I*_G_ of ∼0.2 (g) and 120–430 meV for AH-Gr with *I*_D_/*I*_G_ of ∼0.8 (i).

Figure 1a illustrates the hydrogenation schematics of Gr/SiO_2_ via weak hydrogen plasma. At low temperature (LT), active H radicals bond to the upper side of graphene, while generated protons will permeate the graphene and recombine into H radicals, forming C−H bonds on the bottom side, leading to LH-Gr (scenario I). Noting that, the hydrogenation processes are hugely affected by temperature and concentration of H radicals [18,35,36]. As temperature increases, the formed C−H bonds are easily broken, particularly those on the upper side exposed to the free space. Once hydrogenation gets underway at moderate temperature (MT), C−H bonds on the upper side tend to break while those on the bottom side remain relatively stable because of the sub-nanometer confined space and the accumulated high-concentration of H radicals, leading to formation of AH-Gr (scenario II). If graphene is free-standing, both sides remain non-hydrogenated under MT (scenario III), serving as criteria for the hydrogenation temperature range. At high temperature (HT), C−H bonds on the bottom side of graphene also become unstable, preventing hydrogenation (scenario IV). Therefore, the crucial factors for producing AH-Gr in the confined space should be the hydrogenation temperature range under nondestructive plasma conditions.

Raman spectroscopy is used to characterize C−H bonds in graphene, and the D to G peak intensity ratios (*I*_D_/*I*_G_) serve as indicator of non-*sp*^2^ bonds [38,39]. Figure 1b and c show Raman spectra of free-standing graphene on Cu grid and Gr/SiO_2_, respectively, after undergoing hydrogenation processes at different temperatures. At LT, both exhibit high *I*_D_/*I*_G_ ratios. The reversibility of *I*_D_/*I*_G_ indicates that the D peaks originate from the formed C−H bonds (Fig. S1a and b). As temperature rises, the *I*_D_/*I*_G_ ratios are both reduced, indicating that hydrogenation is suppressed by the elevated temperature. Notably, the D peak in free-standing graphene completely disappears at 350°C, but it still exists in Gr/SiO_2_. Additionally, with prolonged treatment, the D peak in Gr/SiO_2_ becomes more pronounced, while the free-standing graphene remains at a state of basically no-D peak (Fig. 1d and Fig. S1c). The absence of a D peak indicates that free-standing graphene is not hydrogenated. Both sides of the free-standing graphene are exposed to free space, there are no C−H bonds existing on either side. In contrast, the upper side of Gr/SiO_2_ also has free space and should be free of C−H bonds, hence the D peaks should mainly originate from the C−H bonds on the bottom side, i.e. AH-Gr. As temperature exceeds 425°C, D peaks are negligible, indicating no C−H bonds formed. Figure 1e summarizes the normalized *I*_D_/*I*_G_ ratios of free-standing graphene and Gr/SiO_2_ hydrogenated at different temperatures (details in Fig. S1d and e). After undergoing the hydrogenation process at 350–425°C, only Gr/SiO_2_ can be hydrogenated indicated by the distinguishable *I*_D_, while free-standing graphene shows almost no C−H bonds. Consequently, we define the temperature ranges of hydrogenation as LT of 25–350°C for LH-Gr, where the asymmetry of hydrogenated graphene gradually increases with the increase in temperature, MT of 350–425°C and HT of >425°C for AH-Gr and non-hydrogenation, respectively.

Further studies using a scanning tunnelling microscope (STM) show the morphology of AH-Gr with *I*_D_/*I*_G_ ratios of ∼0.2 and ∼0.8, revealing perfect hexagonal lattices, with no upper-side C−H bonds, as shown in Fig. 1f and h. The undamaged lattices without C−H domains further confirm its highly asymmetric feature, and the D peaks should mainly derive from bottom-side C−H bonds. Figure S2 shows typical STM images of graphene hydrogenated at LT, showing numerous upper-side C−H domains, which is the same as the reported surface topography of hydrogenated graphene hydrogenated at LT [19,20,22]. The C−H bonds can be also completely removed by UHV annealing. Notably, the plasma utilized for hydrogenation is too weak to introduce atomic vacancies, as confirmed by the recovered lattices and our reported results [13,35,36]. Moreover, scanning tunnelling spectroscopy (STS) (Fig. 1g and i) show the changed band structure of AH-Gr, with opened bandgaps of 0–30 meV and 120–430 meV for *I*_D_/*I*_G_ of ∼0.2 and ∼0.8, respectively. The STS spectrum of pristine graphene is shown as the blue line in Fig. 1g for comparison. Figure 1j summarizes the bandgap distributions from dozens of spectra (Fig. S3), indicating the changed band structures and probable derivative properties.

Furthermore, we compared the wettability of pristine graphene, LH-Gr and AH-Gr through wetting angle measurements, as shown in Fig. S4. For pristine graphene, the wetting angle is ∼86°. After hydrogenation into LH-Gr (*I*_D_/*I*_G_ = 3.7), the angle decreases to ∼60°, due to increased surface energy [40]. In contrast, AH-Gr consistently exhibits lower wetting angles and higher surface energy compared to LH-Gr with similar *I_D_*/*I_G_* ratios. Additionally, we further investigated the transformation between LH-Gr and AH-Gr using *ex situ* wetting angle measurements. Starting with AH-Gr (*I*_D_/*I*_G_ = 2.4), LT hydrogenation increased the wetting angle from 42° to 57° (*I*_D_/*I*_G_ = 2.6), and then to 73° (*I*_D_/*I*_G_ = 3.5), indicating higher hydrogenation and changes in C−H bond distribution (LH-Gr). The annealing process further transforms LH-Gr back towards AH-Gr, decreasing the wetting angle from 73° to 53° (*I*_D_/*I*_G_ = 2.7). These results demonstrate that hydrogenation temperature can tune the spatial distribution of C−H bonds.

### Utilization factor of permeated protons for AH-Gr

**Figure 2.**
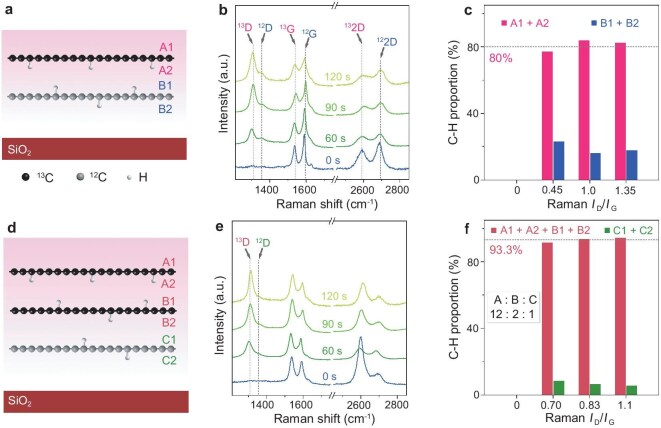
Depth distribution of C−H bonds confirmed through isotopic effects. (a) Schematic of hydrogenating double-layer Gr/SiO_2_, where top and bottom graphene films are constituted by ^13^C and ^12^C, respectively. (b) Raman spectra of double-layer graphene hydrogenated at MT for different durations. The ^13^C−H and ^12^C−H bonds cause the different dividing D peaks, where ^13^D from ^13^C−H is located at 1308 cm^−1^ and ^12^D from ^12^C−H is at 1355 cm^−1^. (c) Distribution of C−H bonds on different sides of graphene, estimated from the relative Raman intensity of the D peak. The proportion of C−H bonds at the bottom side of top graphene reaches ∼80% (A2), where the upper side of top graphene (A1) is negligible. (d) Schematic of hydrogenating triple-layer Gr/SiO_2_, where top, middle and bottom graphene films are constituted by ^13^C, ^13^C and ^12^C, respectively. (e) Raman spectra of triple-layer graphene hydrogenated at MT for different durations. The ^12^D peak from the bottom graphene is very weak. (f) Depth distribution of C−H bonds at different sides. The proportion of C−H bonds at bottom graphene is ∼6.7%. The ratios of C−H bonds at top, middle and bottom graphene are ∼12:2:1.

Next, we estimate the utilization factor of aggregated H radicals for hydrogenating AH-Gr. First, we fabricate a double-layer graphene film consisting of top ^13^C-graphene and bottom ^12^C-graphene, with distinguishable Raman G and D peaks. Subsequently, this double-layer graphene is exposed to H_2_ plasma at MT. The protons permeate into the vdW confined space between ^13^C-graphene and ^12^C-graphene, re-combined H radicals will mainly bond to the bottom side of the top ^13^C-graphene and the upper side of bottom ^12^C-graphene (Fig. 2a). Meanwhile, a few protons permeate ^12^C-graphene and form ^12^C−H bonds at the bottom side. Their Raman spectra with different hydrogenation durations are plotted in Fig. 2b, and the isotopic ^13^C-graphene presents the redshifted ^13^G, ^13^D and ^13^2D peaks. Although the ^13^G intensity is 1/3 weaker than ^12^G, the ^13^D is still twofold stronger than ^12^D. Figure2c plots the relative ratio for ^13^C−H and ^12^C−H bonds, which is normalized by the ratio of *I*_13D_/*I*_13G_ to *I*_12D_/*I*_12G_. We find that the concentration of ^13^C−H is ∼80%, representing the utilization factor for the bottom side hydrogenation of top AH-Gr.

Additionally, we fabricate a triple-layer film consisting of top ^13^C-graphene, middle ^13^C-graphene and bottom ^12^C-graphene. After MT hydrogenation for different durations, we use the ^13^D and ^12^D peaks to estimate the hydrogenation depth through permeating protons, as illustrated in Fig. 2d. Figure 2e displays the Raman spectra of the triple-layer film following varying hydrogenation durations. The weak intensity of ^12^D peak indicates minimal hydrogenation in the bottom ^12^C-graphene, with the concentration of ^12^C−H estimated to be <6.7% (Fig. 2f). The proton permeation depth here is a single or double-layer graphene lattice, consistent with the H_2_ bubble encapsulated by single or double-layer graphene [36].

### Quantification of H/C ratios in AH-Gr

**Figure 3.**
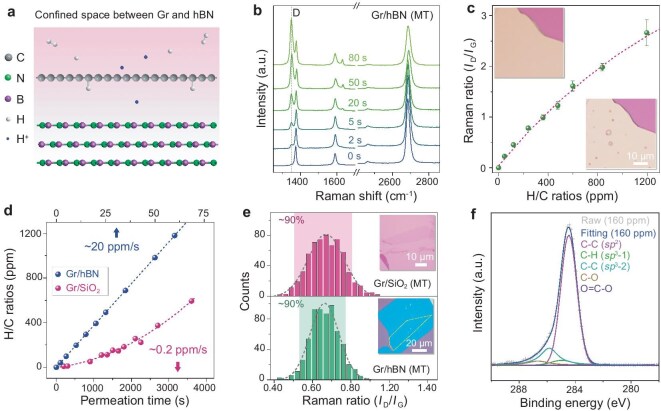
Quantification of H/C ratios in AH-Gr. (a) Schematic of hydrogenating graphene in the vdW confined space between graphene and hBN. The confined space is dense so wastes few active H radicals for hydrogenation. (b) Raman spectra of Gr/hBN with different hydrogenation time periods at MT, showing a rapid intensity increase of D peak. (c) Correlation between Raman *I*_D_/*I*_G_ and H/C ratios. Insets are the optical images of graphite flakes before (top) and after (bottom) proton permeation at HT under the same proton conditions. The circular dots in graphite are formed by H_2_ bubbles in the permeated protons. H_2_ bubbles formed in graphene lattices prolong the permeation duration at HT, but there is no bubble formed at MT. (d) Correlation between H/C ratios and hydrogenation time for graphene on different substrates. The H/C ratio of Gr/hBN increases more rapidly than that of Gr/SiO_2_. (e) Statistic histograms of Raman *I*_D_/*I*_G_ for Gr/SiO_2_ (top) and Gr/hBN (bottom) hydrogenated at MT. AH-Gr on hBN shows more homogeneous distribution for H/C ratio. Insets are their corresponding optical images before undergoing hydrogenation. (f) XPS of AH-Gr film on SiO_2_/Si with H/C ratio of 160 ppm. The sub-peak indicates the existence of *sp*^3^ C−H component.

We utilize exfoliated hBN as a denser substrate to create homogeneous vdW confined spaces, where recombined H radicals are assumed to efficiently form C−H bonds [35,36,41,42]. Figure 3a illustrates the schematic of AH-Gr on hBN, the distance of vdW confined space is typically 0.34–0.65 nm for various 2D materials [10,33,43,44]. After transferring monolayer graphene onto few-layer hBN [45], we perform hydrogenation, Gr/hBN exhibits a significantly enhanced hydrogenation effect compared to Gr/SiO_2_, and with an extended temperature threshold for AH-Gr up to 460°C (Fig. S5).

To quantitatively estimate the H/C ratios of AH-Gr, we establish an analogy approach. Given that hBN and graphene exhibit lower permeability to protons compared to amorphous SiO_2_ [33], ∼80% of the permeated protons are utilized for generating AH-Gr. This allows us to calculate the C−H ratios via the dose of the permeated protons following the equation H/C = *Γ*_P_  *×* τ/*n*_C_, where *Γ*_P_ is the permeating rate of proton through graphene and is estimated to be ∼940 s^−1^μm^−2^ under the same plasma condition [36], τ is the proton permeation time, and *n*_C_ is the carbon atom density in graphene of 3.82 × 10^19^ m^−2^.

Figure 3b and c plot the typical Raman spectra of Gr/hBN after different permeation times and the extracted *I*_D_/*I*_G_ versus calculated H/C ratios, respectively. After 5 s of hydrogenation, the *I*_D_/*I*_G_ ratio reaches 0.6, corresponding to an H/C ratio of 98 ppm, with the average distance between neighboring C−H bonds (*L*_H_) being ∼16.3 nm. This distance aligns closely with Raman analysis [38], but is slightly larger than that established by e-beam irradiated HSQ [28]. We then compare the correlation between H/C ratios and hydrogenation duration for Gr/hBN and Gr/SiO_2_. Notably, AH-Gr on both substrates with the same H/C ratios exhibit identical *I*_D_/*I*_G_, confirmed by transferring the same AH-Gr films to SiO_2_/Si and hBN (Fig. S6). This indicates that Raman *I*_D_/*I*_G_ for AH-Gr only derives from H/C ratios, and are irrelevant to the substrate. Figure 3d shows that the average hydrogenation rate is ∼20 ppm/s for Gr/hBN, significantly higher than the 0.2 ppm/s for Gr/SiO_2_. The two orders magnitude difference indicating a narrower space of Gr/hBN with denser H radicals. The higher *I*_D_/*I*_G_ ratio for AH-Gr/SiO_2_ requires a longer hydrogenation duration (Fig. 1e). Additionally, the vdW confined space enhances the homogeneity of AH-Gr, as confirmed in Fig. S7. Figure 3e plots the *I*_D_/*I*_G_ spatial distributions of AH-Gr/SiO_2_ and AH-Gr/hBN. Although both exhibit similar H/C ratios with an average *I*_D_/*I*_G_ ratio of 0.67, the distribution for Gr/SiO_2_ is notably broader, with ∼90% of counts between 0.52 and 0.79, whereas Gr/hBN is more homogeneous with ∼90% of counts between 0.55 and 0.76.

X-ray photoelectron spectroscopy (XPS) is employed to analyze the elemental composition and chemical states. Figure 3f shows the XPS spectra of AH-Gr with H/C ratio of 160 ppm, with distinct subpeaks for different components. The C−H bonds (*sp*^3^-1) peak is located at 285.0 eV, C−C bonds (*sp*^2^) of graphene at 284.5 eV. Through peak differentiation, the C−H (*sp*^3^-1) component is extracted to estimate the H/C ratio. The XPS spectra of as-transfer graphene are shown in Fig. S8, where no C−H (*sp*^3^-1) peak is visible. Additional component peaks, like C−C (*sp*^3^-2) at 285.9 eV, C−O at 286.7 eV and O=C−O at 288.9 eV correspond to the quaternary carbon, methoxy group and carboxyl group, respectively, which may originate from the residual polymethyl methacrylate (PMMA) [46].

### Electrical and magnetic properties of AH-Gr with different H/C ratios

**Figure 4.**
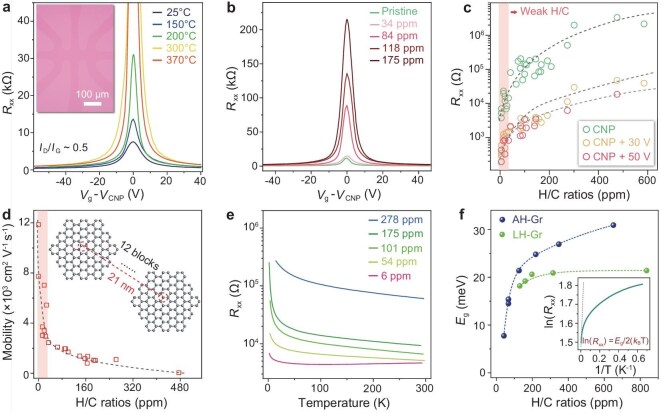
Electrical performances of AH-Gr films. (a) *R*_xx_ of the hydrogenated Gr/SiO_2_ as a function of *V*_g_ − *V*_CNP_. The films are hydrogenated at different temperatures but with the same Raman *I*_D_/*I*_G_ of 0.5. Inset is the typical optical image of a hydrogenated graphene Hall device. (b) *R*_xx_ of AH-Gr with different H/C ratios as a function of *V*_g_—*V*_CNP_. (c) Statistical distribution of *R*_xx_ of AH-Gr with various H/C ratios. *R*_xx_ values are collected from the CNP (green), light doped state (yellow, *V*_CNP_ + 30 V) and heavy doped state (red, *V*_CNP_ + 50 V). The resistances increase sharply among the weak hydrogenation range, highlighted by light red. (d) Statistics of carrier mobility of AH-Gr with different H/C ratios. AH-Gr films with low H/C ratios behave like semi-metals while maintaining high mobility, highlighted by light red. Inset is the schematic of the *L*_H_, which is ∼21 nm for AH-Gr with an H/C ratio of 40 ppm. (e) Temperature-dependent *R*_xx_ of AH-Gr with different H/C ratios. *R*_xx_ is collected at CNP. Most AH-Gr films behave like a typical semiconductor. (f) Extracted *E*_g_ of AH-Gr and LH-Gr with different H/C ratios. LH-Gr is prepared at 200°C [47]; the dashed line is a guideline to the trend. Inset is the fitted *E*_g_ from the temperature-dependent *R*_xx_.

To investigate the impact of hydrogenated graphene with varying C−H bond distributions, we perform electrical transport measurements on films hydrogenated at different temperatures, all with an identical Raman *I*_D_/*I*_G_ ratio of ∼0.5. Figure 4a and Fig. S9a plot gate voltage (*V*_g_) dependent four-probe longitudinal resistances (*R*_xx_). All the electrical transport curves remain bipolar, the maximum *R*_xx_ at charge neutrality point (CNP) increasing with hydrogenation temperature, with values of 7.5, 14, 31,104 and 107 kΩ for 25,150, 200,300 and 370°C, respectively. This indicates a transformation of samples from LH-Gr to AH-Gr. Notably, graphene films hydrogenated at 300°C have similar resistance to AH-Gr prepared at 370°C, indicating that hydrogenation at 300°C approximates AH-Gr.

Figure 4b shows that *R*_xx_ of AH-Gr increases with H/C ratios. At H/C ratio of 175 ppm, *R*_xx_ is 216 kΩ at CNP, about 20-fold higher than pristine graphene. This substantial increase should be attributed to the C−H scattering sites and the enhanced inversion asymmetry. Comparatively, for AH-Gr with 34 ppm H/C ratios, *R*_xx_ demonstrates a slight increase of 15%, indicating weak hydrogenation, where partial graphene exhibits the properties of a semiconductor. Figure 4c presents *R*_xx_ statistics of AH-Gr with various H/C ratios at for CNP (green circles), moderate doping (yellow circles, *V*_CNP+30 V_) and heavy doping (red circles, *V*_CNP+50 V_). Generally, *R*_xx_ at CNP is ∼2 orders of magnitude higher than under both moderate doping and heavy doping. Moreover, for an H/C ratio of 474 ppm, *R*_xx_ at CNP reaches 3.4 MΩ, ∼300 times higher than pristine graphene. A significant trend shows that *R*_xx_ rapidly increases from 0 to 40 ppm H/C ratio, indicating a weak C/H region with coexisting semi-metal and semiconductor regions. Furthermore, higher ratios indicate bandgap opening in all carbon lattices.

Next, we calculate the carrier mobility of AH-Gr with different H/G ratios, summarized in Fig. 4d. Carrier mobilities decrease with increasing H/C ratio, consistent with the reduced conductivity. It drops sharply from ∼12 000 to ∼2000 cm^2^V^−1^s^−1^ within the weak hydrogenation degree, then gradually declines. The AH-Gr with an H/C ratio of ∼174 ppm (STS-measured bandgap of 240 meV) maintains high mobility ∼1000 cm^2^V^−1^s^−1^, while with ∼480 ppm, mobility decreases to ∼40 cm^2^V^−1^s^−1^. Notably, the average distance between the neighboring C−H bonds (*L*_H_) is calculated to be ∼21 nm for AH-Gr with an H/C ratio of 40 ppm, which may be the sparsest distance of hydrogen distribution that can open a bandgap for surrounding carbon lattices.

Figure 4e shows the temperature-dependent resistance (*R-T*) measurements of AH-Gr. For AH-Gr with weak H/C ratio of 6 ppm, *R*_xx_ at CNP drops from 110 to 1.5 K, then increases from 110 to 295 K, similar to pristine graphene (Fig. S9b–e). For higher H/C ratios (54–278 ppm), *R*_xx_ decreases with rising temperature, indicating typical semiconductor behavior. We further fit their bandgaps from the Arrhenius equation *R*_xx_ = exp[*E*_g_/(2*k*_B_*T*)], where *E*_g_ is the bandgap, *k*_B_ is the Boltzmann constant and *T* is the temperature [21], as plotted in Fig. 4f, marked by blue dots, with the dashed line being a guideline to the trend. The fitted *E*_g_ is ∼1 order magnitude smaller than the STS value, likely due to the thermally excited carriers and substrate-induced pseudo-bandgap [48]. By comparing LH-Gr hydrogenated at 200°C, as redrawn in Fig. 4f by green dots, AH-Gr opens a larger bandgap while they have the same hydrogenation concentration. The *R-T* measurements of LH-Gr are shown in Fig. S9f.

**Figure 5.**
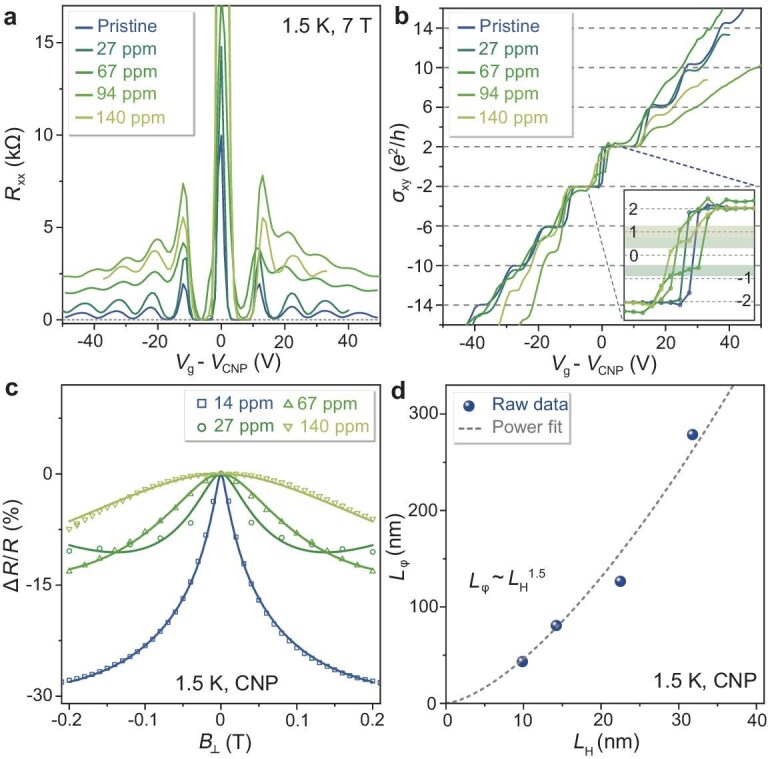
Magnetotransport measurement of AH-Gr on SiO_2_/Si. (a, b) *R*_xx_ and *σ*_xy_ of the AH-Gr with different H/C ratios as a function of *V*_g_—*V*_CNP_ at 1.5 K under *B*_┴_ of 7 T. The horizontal dashed lines in (b) are the guidelines of the Hall plateaus. The Hall plateaus at half-integer are becoming deviated as the H/C ratios increase. Inset of (b) shows the enlarged region of *σ*_xy_ plateaus between −2 and 2. (c) The change rates of MR with B^⊥^. The MR of AH-Gr with different H/C ratios is collected at CNP and 1.5 K. The results are fitted with solid lines by the WL theory. (d) Relationship between *L*_φ_ and *L*_H_, which are approximately fitted by the power function of *L*_φ_ = *L*_H_^1.5^.

Magnetotransport measurements of AH-Gr films with varying C−H bonds monitor the evolution changes of the quantum Hall effect (QHE) [49]. Figure 5a and b plot *R*_xx_ and Hall conductivity (*σ*_xy_) of AH-Gr films under out-of-plane magnetic field (*B*_┴_) of 7 T at 1.5 K. The *R*_xx_ in as-transferred graphene film fall to zero, and *σ_xy_* reach ± 2 *e*^2^/*h* plateaus near *V*_g_ = ±7 V, corresponding to Landau filling factors *ν* = ±2. Meanwhile, only AH-Gr with weak H/C ratio (34 ppm) shows zero *R*_xx_, and *σ_xy_* remains plateau shaped at *ν* = ±6. For other AH-Gr films, *R*_xx_ cannot reach zero and *σ_xy_* plateaus mismatch filling factors, because of the hydrogen-induced broadening of Landau levels [49]. Additionally, the *σ_xy_* of AH-Gr with H/C ratios ranging from 84–175 ppm show slight slopes near CNP, corresponding to the *R*_xy_ peaks (Fig. S9g). These dramatically changed peaks are expected to originate from the disruption of topological edge states induced by C−H bond-induced disorders at CNP [50]. Moreover, weak H/C ratio AH-Gr (34 ppm) still shows topological edge states, indicating interconnections between semimetal and semiconductor regions.

Magnetoresistance (MR) of AH-Gr at CNP with different H/C ratios is performed under weak *B*_┴_ at 1.5 K. Figure 5c shows that MR decreases with enlarged *B*_┴_, and the change rates of MR with *B*_┴_, that is (Δ*R*)/*R* = [*R*(*B*_┴_)—*R*(*B*_┴_ = 0)/*R*(*B*_┴_ = 0)]. Using weak localization (WL) theory [51], we fitted MR to obtain a phase coherence length (*L*_φ_) of AH-Gr, with relationship to *L*_H_ plotted in Fig. 5d. *L*_φ_ of AH-Gr increases from 34 to 233 nm as *L*_H_ increases from 13 to 41 nm, and they have an approximate power function dependence of *L*_φ_ ∼ *L*_H_^1.5^. The reason is that C−H bonds can act as scattering sites causing charge carrier de-coherence [52].

## CONCLUSIONS

In summary, we develop a confined space assisted production of AH-Gr via proton permeation at an MT of 350–425°C. LT hydrogenation leads to LH-Gr, while HT terminates hydrogenation. AH-Gr films are defect-free with opened bandgaps. HT annealing can remove all the formed C−H bonds, recovering their hexagonal lattices. The H/C ratios can be quantitatively controlled by calculating the permeated protons in the vdW confined space between graphene and hBN. AH-Gr on hBN shows a higher hydrogenation rate of 20 ppm/s, which is 2 orders of magnitude greater than on amorphous SiO_2_. Due to larger structural asymmetry, AH-Gr exhibits higher resistance and larger bandgap than LH-Gr at similar hydrogenation degree. When H/C ratios exceed 40 ppm, AH-Gr changes from semi-metal to semiconductor. Magnetotransport measurements indicate that hydrogenation-induced disruption affects the QHE and reduces the *L*_φ_. This study re-examines the production and features of hydrogenated graphene, expands the applications of vdW confined space, and presents a quantitative hydrogenation approach for AH-Gr with controlled spatial distribution.

## Supplementary Material

nwaf067_Supplemental_File
