# Supplementary material for: Realization of highly asymmetric hydrogenated graphene in the van der Waals confined space
Source: Natl Sci Rev. 2025 Feb 22;12(4):nwaf067. doi: 10.1093/nsr/nwaf067 (PMC11932338; doi:10.1093/nsr/nwaf067)
Supplement: nwaf067_Supplemental_File [file nwaf067_supplemental_file.pdf]

Supporting Information for

Realization of highly asymmetric hydrogenated graphene in the van der Waals confined space

Xianlei Huang[1,†], Hang Zheng[1,†], Weilin Liu[1,†], Li Zhu[1,†], Guowen Yuan[1,*], Jie Xu[1,2], Kaiyuan Wang[1], Lei Wang[1], Shao-Chun Li[1,*], and Libo Gao[1,*]

[1] National Laboratory of Solid State Microstructures, Jiangsu Key Laboratory for Nanotechnology, School of Physics, Collaborative Innovation Center of Advanced Microstructures, Nanjing University, Nanjing, 210008, China.

[2] Anhui Provincial Key Laboratory of Magnetic Functional Materials and Devices, School of Materials Science and Engineering, Anhui University, Hefei, 230601, China.

*Corresponding authors. E-mails: gwyuan@nju.edu.cn; scli@nju.edu.cn; lbgao@nju.edu.cn

[†]Equally contributed to this work.

EXPERIMENTAL AND METHODS

Preparation of graphene. Pristine graphene flakes are exfoliated on SiO_2/Si wafer via traditional micromechanical exfoliation method, and then all the flakes are annealed under high vacuum (<10^{-5} Pa) at 350 ℃ for 30 min to remove polymer residues. Wafer-sized graphene films are grown by proton-assisted chemical vapor deposition (CVD) method as reported before, and the typical growth parameters are as follows: growth temperature of 650 ℃, pressure of 6 Pa, CH_4/H_2 ratio of 1:20, inductively coupled plasma (ICP) power of 15 W and growth time of 5 min, growth substrates of 800 nm Cu-Ni(111) alloy (90% Cu + 10% Ni) films on c-plane sapphire. To simplify writing, we use Cu(111) instead of Cu-Ni(111) alloy in main content. The ^{13}C graphene films are grown with the same parameters except the precursor of $^{13}CH_4$.

Transfer of graphene. Wafer-sized CVD grown graphene films on SiO_2/Si or transmission electron microscope (TEM) grids are transferred by polymethylmethacrylate (PMMA) through the traditional wet method and a proton-assisted cleaning process. Exfoliated Gr/hexagonal boron nitride (hBN) heterostructures is fabricated using a typical vdW pick-up technique (hBN is purchased from XFNANO). CVD-grown Gr/hBN is fabricated by transferring CVD-grown graphene film onto the exfoliated hBN/SiO_2/Si through the traditional wet method and a proton-assisted cleaning process.

Hydrogenation of graphene. For highly asymmetric hydrogenated graphene (AH-Gr), typical treatment parameters are as follows: ICP as plasma source, pressure of 5 Pa, pure H_2, plasma power of 10 W, substrate temperature of 350 - 450 ℃, and treatment time of 60 - 3600 s. For lowly asymmetric hydrogenated graphene (LH-Gr), all the treatment parameters are the same except that the sample temperature is set to be lower than 350 ℃. Sample temperature higher than 450 ℃ will terminate the hydrogenation. For hydrogenation in Figure 1b,c, the treatment time is set to be 300 s.

Structural characterizations. Raman spectroscopy is carried out by a WITec/alpha 300 R confocal microscope using a 100× objective lens under atmospheric environment. The wavelength for the excitation laser is 532 nm (2.33 eV) with the power below 2 mW to avoid heating, and the spot size for the 100× objective lens is estimated to be 500 nm, which is the spatial resolution for Raman mapping. AFM measurements are carried out by a Bruker Dimension Fastscan system at tapping mode under atmospheric environment. STM and STS differential conductance (dI/dV) point spectra

of AH-Gr films on SiO_2/Si are performed under UHV (base pressure <10^{-9} Pa) at 77 K. They are measured in constant-height mode using standard lock-in techniques (f=773.1 Hz), the bias voltage in Figure 1f and 1h are 100 mV and 1.0 V respectively, and both the tunnelling current is 100 pA. XPS is performed using a PHI 5000 X-ray photoelectron spectrometer equipped with a monochromatic using Al Kα radiation ($h\nu$ = 1486.6 eV), and the peaks are fitted by the approximation of Lorentzian-Gaussian mixed function.

Device fabrication and electrical measurements. Graphene on SiO_2/Si is patterned into the geometry of Hall bar using the electron beam lithography (EBL) or ICP reactive ion etching (ICP-RIE, ULVAC CE-300I) through a Mo shadow mask with as-fabricated pattern. ICP-RIE with O_2 plasma is applied to remove the exposed graphene area, and gold wires are bonded on the terminals of fabricated Hall bars by manual operation. Electrical and magneto transport measurements are mainly performed in a ^{4}He cryostat with a superconducting magnet (Oxford Teslatron 8 T) with base temperature of 1.5 K. For four-probe electrical measurements, we mainly use the instruments of lock-in amplifier (Stanford SR830, f = 3.3 Hz) as source and meter. For the sample with ultra-high resistance, the two-probe electrical measurements are performed by using *d.c.* SourceMeter (Keithley 2450) and nanovoltmeter (Keithley 2182A). All the gate voltages are applied by an individual SourceMeter (Keithley 2450).

Calculation methods of L_H and L_φ. The average distance between the neighboring C-H bonds (L_H) is derived by H/C ratios with $L_H = (n_C \times \text{H/C})^{-1/2}$, where n_C is the carbon atom density in graphene of 3.82×10^{19} m^{-2}, H/C is the atomic ratio of hydrogen to carbon. The phase coherence length (L_φ) is fitted by

$$\frac{\Delta R}{R} = \frac{-e^2 R_s}{\pi h}\left[F\left(\frac{\tau_B^{-1}}{\tau_\varphi^{-1}}\right) - F\left(\frac{\tau_B^{-1}}{\tau_\varphi^{-1} + 2\tau_{\text{inter}}^{-1}}\right) - 2F\left(\frac{\tau_B^{-1}}{\tau_\varphi^{-1} + \tau_{\text{inter}}^{-1} + \tau_{\text{intra}}^{-1}}\right)\right] \quad (1)$$

$$L_\varphi^2 = D\tau_\varphi \quad (2)$$

Here R_s is the sheet resistance of graphene, $F(x) = \ln(x) + \Psi(1/2+1/x)$, where Ψ is the digamma function, τ_φ is the phase coherence time, τ_{inter} is the intervalley scattering time, τ_{intra} is the intravalley scattering time, $\tau_B^{-1} = 4eDB/\hbar$, $D = 0.5v_F^2(\tau_{\text{inter}}^{-1} + \tau_{\text{intra}}^{-1})^{-1}$, and v_F is the Fermi velocity of ~10^6 m/s. The τ_{intra} is related to the charge impurities and we assign the value of ~300 fs, τ_φ and τ_{inter} are obtained

from the fitting.

Note: Additional experimental data.

We compare the wettability of pristine graphene, LH-Gr and AH-Gr in Figure S4a,b, and their wetting angles are measured to be ~86°, 60° - 86° and 30° - 60°, indicating the maximum structural asymmetry in AH-Gr causes the higher surface energy and hydrophilicity. We continue to *ex situ* measure the evolution of wetting angles in Figure S4c,d. The wetting angle of AH-Gr (I_D/I_G ~2.4) is 42°, and it increases to 57° after the shorter hydrogenation at LT. At this moment, the I_D/I_G ratio is nearly unchanged, and the AH-Gr should be changed into LH-Gr. After prolonging the LT hydrogenation duration, the wetting angles are increased to ~72° and thereafter keep unchanged. If we slightly decrease the I_D/I_G ratio of LH-Gr by UHV annealing, the wetting angle can be gradually reduced. All these changes of wettability in graphene confirm that the spatial distribution of C-H bonds can be tuned by the hydrogenation temperature.

The vdW confined space can also extend the temperature threshold for highly asymmetric hydrogenation (Figure S5a-c). For instance, the I_D/I_G ratio of Gr/hBN increases to 2.0 only after 60 s hydrogenation, whereas the ratio for Gr/SiO_2 still remains near zero. Moreover, the vdW confined space can also extend the temperature threshold for highly asymmetric hydrogenation. The upper limit temperature for AH-Gr/SiO_2 is normally 425 ℃, but the temperature for AH-Gr/hBN will reach 460 ℃ (Figure S5d-f). The AH-Gr/hBN can also be completely recovered after the vacuum annealing (Figure S5g).

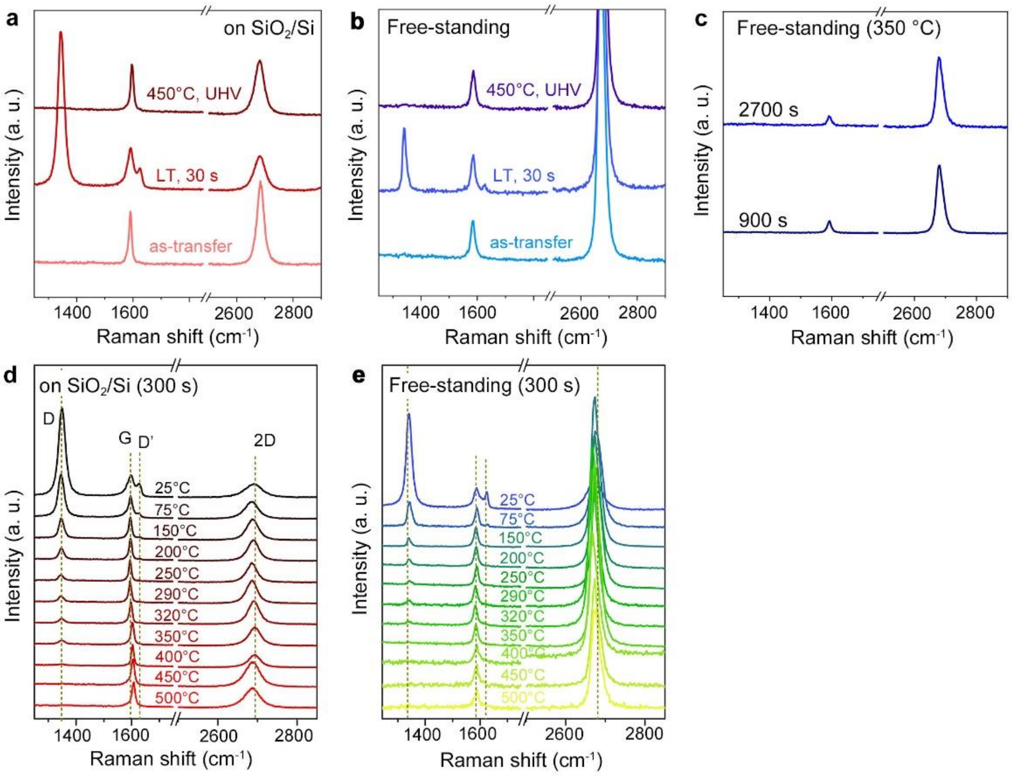

Figure S1. Additional Raman spectra of hydrogenated graphene. a, Reversibility of LH-Gr on SiO_2/Si at LT (25°C), the C-H bonds can be completely removed after the UHV annealing at 450°C. **b**, Reversibility of free-standing LH-Gr at LT (25°C), the C-H bonds can be completely removed after the UHV annealing at 450°C. **c**, Typical Raman spectra of free-standing graphene after hydrogenation at MT (350°C) for 900 s and 2700 s, there are no apparent D peaks. **d**, Detailed Raman spectra of Gr/SiO_2 hydrogenated at different temperatures for 300 s, the hydrogenation occurs at the temperature below 350°C. **e**, Detailed Raman spectra of free-standing graphene hydrogenated at different temperatures for 300 s, the hydrogenation occurs at the temperature below 320°C.

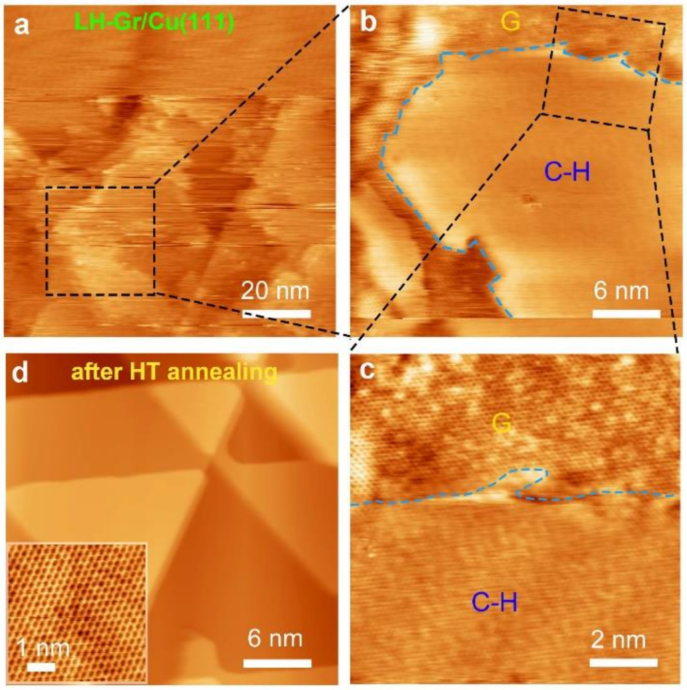

Figure S2. STM images of LH-Gr on Cu(111). a-c, STM images of LH-Gr on Cu(111) substrates at LT (25°C). The plenty of C-H domains are agglomerated on the top side of LH-Gr. **d**, STM image of LH-Gr on Cu(111) after UHV annealing. The agglomerated C-H domains are completely removed, and the inset shows the hexagonal lattice of dehydrogenated graphene is perfect and there are no vacancy defects.

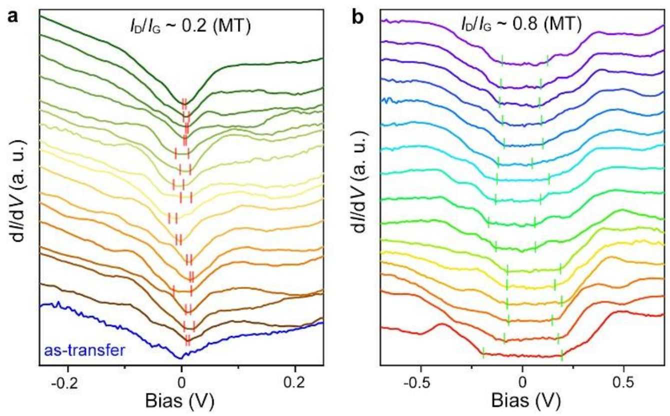

Figure S3. Detailed STS spectra of AH-Gr films on SiO_2/Si. a, STS spectra of AH-Gr with Raman I_D/I_G of ~0.2, all AH-Gr films emerge the opened bandgaps up to 30 meV. The pristine graphene with zero bandgap is also plotted for comparison. **b**, STS spectra of AH-Gr with I_D/I_G of ~0.8, all AH-Gr films emerge the opened bandgaps ranging from 120 to 430 meV.

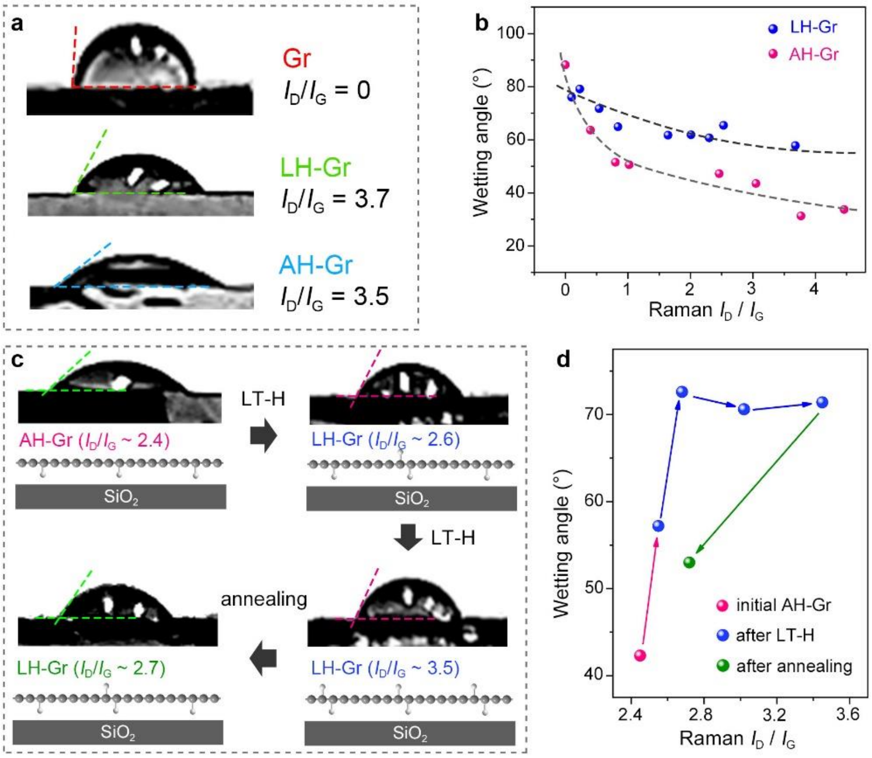

Figure S4. Wetting angle evolution of hydrogenating graphene films. a, Cross-section photos of the water drop on pristine graphene, LH-Gr with I_D/I_G ~ 3.7 and AH-Gr films with I_D/I_G ~ 3.5. Their wetting angles are different. **b**, Statistical wetting angles of AH-Gr and LH-Gr with different I_D/I_G. The wetting angles of AH-Gr are smaller than the LH-Gr with the same Raman I_D/I_G. **c**, *ex situ* wetting angle evolution from AH-Gr of I_D/I_G ~ 2.4, LH-Gr of I_D/I_G ~ 2.6 (LT hydrogenation), LH-Gr of I_D/I_G ~ 3.5 (LT hydrogenation) to LH-Gr of I_D/I_G ~ 2.7 (UHV annealing). **d**, Corresponding wetting angle evolution, the double-sided hydrogenation will increase the wetting angles.

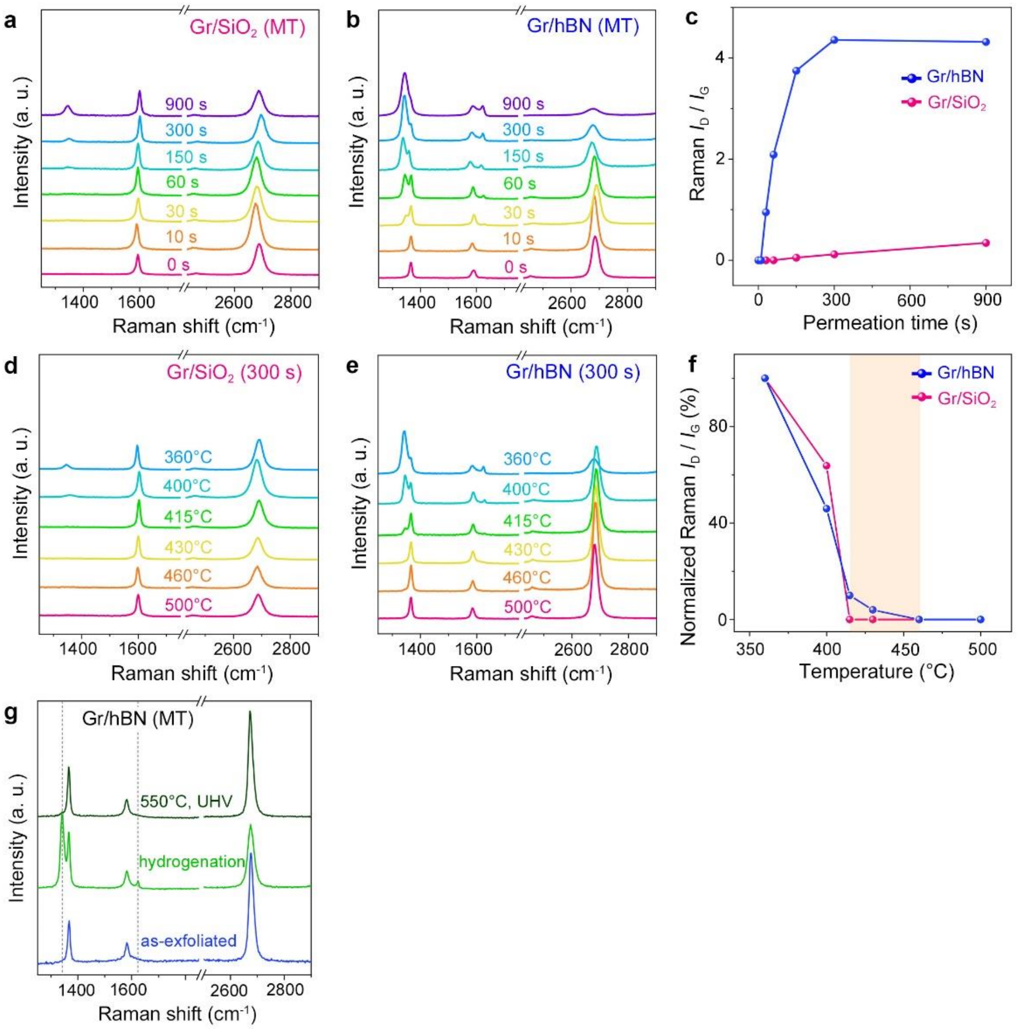

Figure S5. Detailed Raman spectra of AH-Gr on SiO_2/Si and hBN. **a**-**b**, Raman spectra of hydrogenating Gr/SiO_2 (**a**) and Gr/hBN (**b**) at MT with different times. **c**, Extracted Raman I_D/I_G with different treatment times at MT. **d**-**e**, Raman spectra of hydrogenating Gr/SiO_2 (**d**) and Gr/hBN (**e**) at different temperatures for 300 s. **f**, Normalized Raman I_D/I_G with different temperatures for 300 s. Graphene on hBN could be hydrogenated at a higher temperature than on SiO_2. **g**, Reversibility of AH-Gr to pristine graphene on hBN via UHV annealing, the D peak and corresponding C-H bonds can be fully removed.

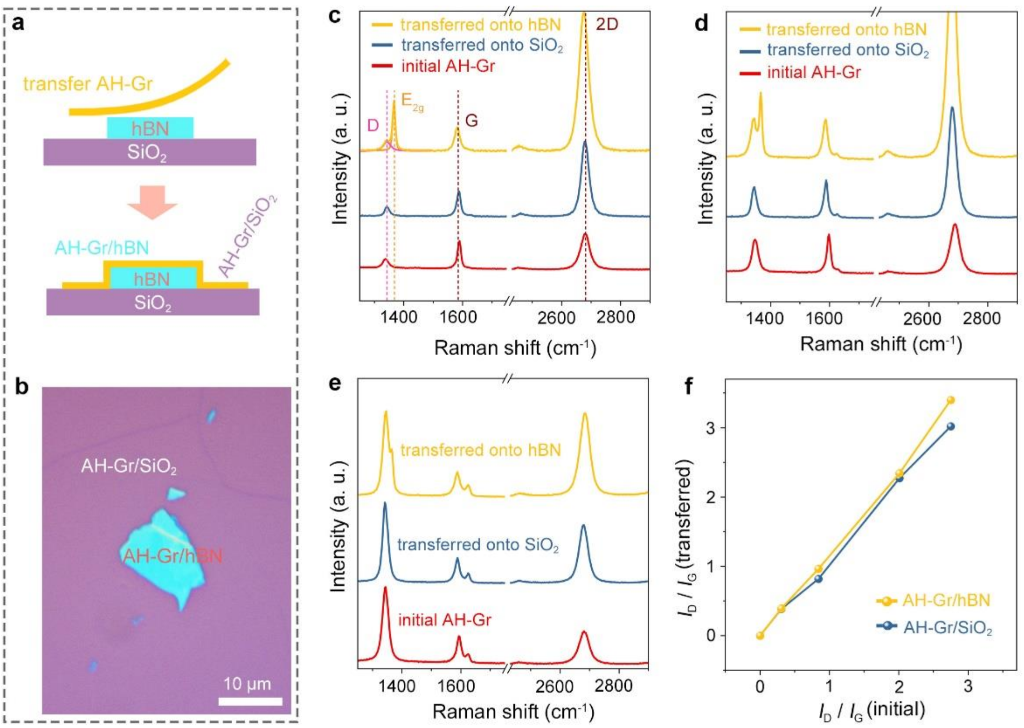

Figure S6. Stability of C-H bonds in AH-Gr. a-**b**, Schematics (**a**) and optical image (**b**) of fabricated AH-Gr/hBN and AH-Gr/SiO_2, where hBN flakes are pre-exfoliated on SiO_2/Si. **c**-**e**, Raman spectra of AH-Gr with different I_D/I_G after transferring on hBN and SiO_2/Si. **f**, Relationship between Raman I_D/I_G of AH-Gr before and after being transferred. Their I_D/I_G values approximately keep the same, indicating the C-H bonds in AH-Gr are stable and not affected by the substrate.

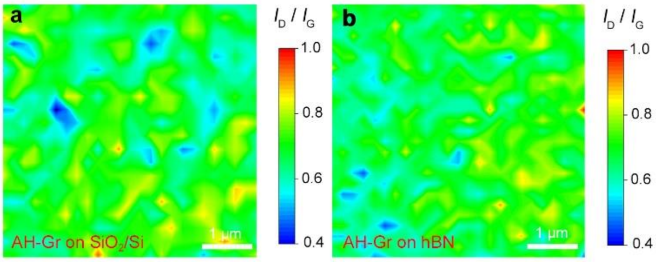

Figure S7. Homogeneity of C-H bonds in different vdW confined spaces. a, b, Raman mapping of I_D/I_G for AH-Gr on SiO_2/Si (**a**) and hBN (**b**), respectively. The vdW confined space between graphene and hBN are denser and more homogenous than that between graphene and amorphous SiO_2, resulting in more uniformly distributed C-H bonds for Gr/hBN.

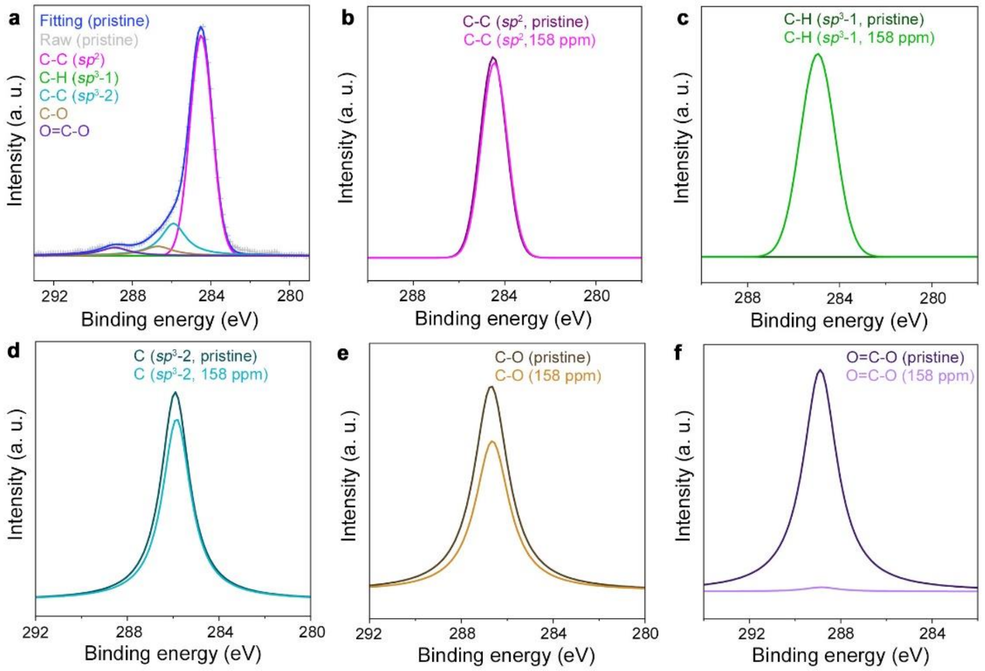

Figure S8. XPS peak analysis of AH-Gr. a, XPS spectrum of the C 1*s* core-level of pristine graphene on SiO_2/Si. **b**-**f**, Comparison of C 1*s* core-level XPS component between pristine graphene and AH-Gr (158 ppm), including C (284.5 eV, C-C, sp^2) of graphene (**b**), C (285.0 eV, sp^3-1, C-H) of graphene (**c**), C (285.9 eV, sp^3-2, quaternary carbon) of polymethylmethacrylate (PMMA) (**d**), C-O (286.7 eV, methoxy group) of PMMA (**e**), and O=C-O (288.9 eV, carboxyl group) of PMMA (**f**). The subpeak for sp^3-1 at 285 eV is highly different between pristine graphene and AH-Gr, indicating the successful hydrogenation.

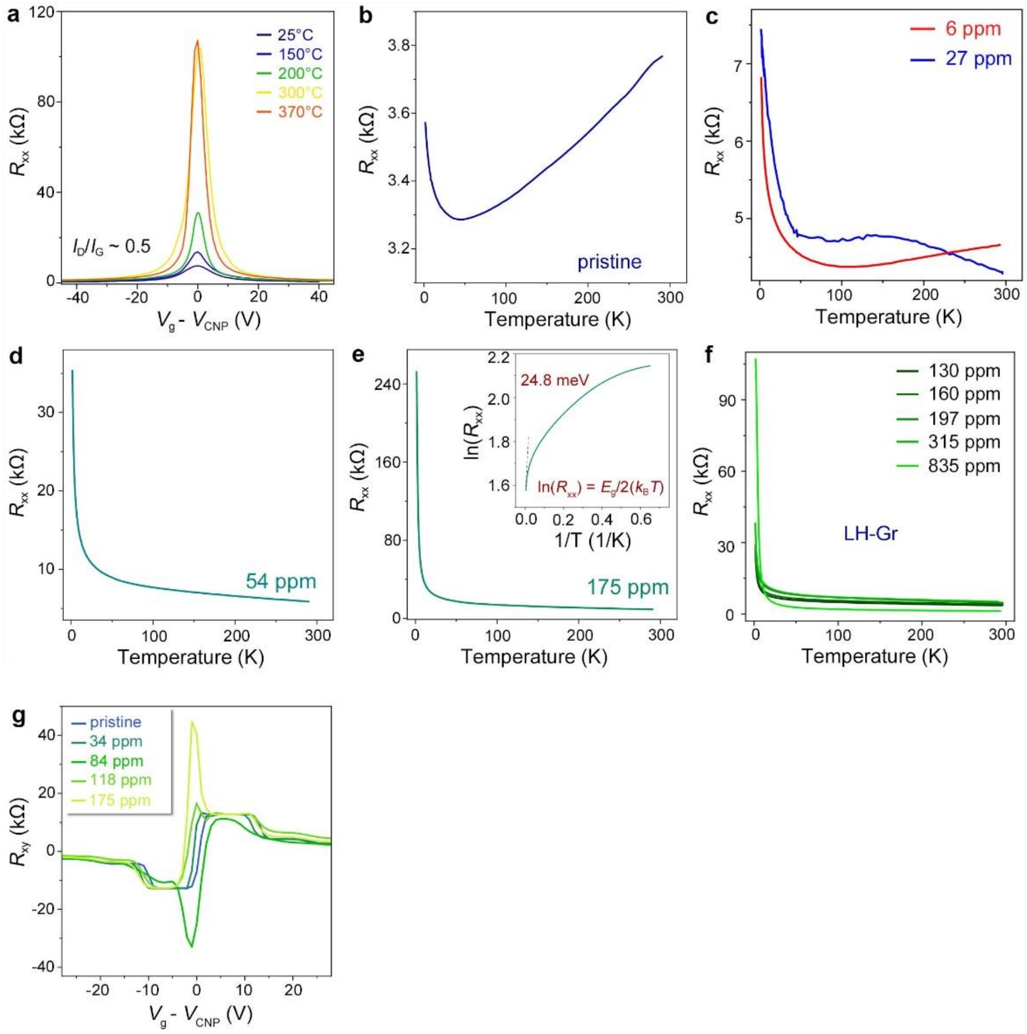

Figure S9. Additional electrical measurements of AH-Gr on SiO_2/Si. a, Full viewed electrical transport results of the graphene hydrogenated at different temperatures, which are identical to the main Fig. 4a. **b**-**e**, Temperature dependent R_{xx} measured at CNP of pristine graphene (**b**), AH-Gr with H/C ratios of 6 and 27 ppm (**c**), AH-Gr with H/C ratio of 54 ppm (**d**), and AH-Gr with H/C ratio of 175 ppm (**e**). Inset of **e** is the fitted bandgap for the 175 ppm AH-Gr. **f**, Temperature dependent R_{xx} measured at CNP of LH-Gr with different H/C ratios, where the H/C ratio of 197, 315, and 835 sccm are redrawn from our published work of main Reference [47]. **g**, Magnetotransport measurements for Hall resistance (R_{xy}) of AH-Gr with different H/C ratios at 1.5 K under $B_\perp$ of 7 T, corresponding to the σ_{xy} of the main Fig. 5b. The dramatically altered R_{xy} peaks are the result of C-H bond induced disorders.
